# The effect of delegation of therapy to allied health assistants on patient and organisational outcomes: a systematic review and meta-analysis

**DOI:** 10.1186/s12913-020-05312-4

**Published:** 2020-06-03

**Authors:** David A. Snowdon, Beth Storr, Annette Davis, Nicholas F. Taylor, Cylie M. Williams

**Affiliations:** 1 Professional Academic Unit, Peninsula Health, Monash University, Frankston, VIC 3199 Australia; 2 Department of Physiotherapy, Peninsula Health, Monash University, Frankston, VIC 3199 Australia; 3 Allied Health Workforce Innovation Strategy Education Research (WISER) unit, Monash Health, Clayton, VIC 3168 Australia; 4 Allied Health Clinical Research Office, Eastern Health, Box Hill, 3128 Australia; 5 College of Science, Health and Engineering, La Trobe University, Bundoora, 3083 Australia

**Keywords:** Allied health assistant, Allied health professional, Delegation, Therapy, Patient outcomes, Safety

## Abstract

**Background:**

Allied health assistants (AHAs) are support staff who complete clinical and non-clinical tasks under the supervision and delegation of an allied health professional. The effect of allied health professional delegation of clinical tasks to AHAs on patient and healthcare organisational outcomes is unknown. The purpose of this systematic review was to investigate the effect of allied health professional delegation of therapy to AHAs on patient and organisational outcomes.

**Methods:**

A systematic review and meta-analysis was conducted. Databases MEDLINE (Ovid), Embase (Ovid), Informit (all databases), Emcare (Ovid), PsycINFO (Ovid), Cumulative Index to Nursing and Allied Health Literature [CINAHL] (EbscoHost) and the Cochrane Database of Systematic Reviews were searched from earliest date available. Additional studies were identified by searching reference lists and citation tracking. Two reviewers independently applied inclusion and exclusion criteria. The quality of the study was rated using internal validity items from the Downs and Black checklist. Risk ratios (RR) and mean differences (MD) were calculated for patient and organisational outcomes. Meta-analyses were conducted using the inverse variance method and random-effects model.

**Results:**

Twenty-two studies met the inclusion criteria. Results of meta-analysis provided low quality evidence that AHA supervised exercise in addition to usual care improved the likelihood of patients discharging home (RR 1.28, 95%CI 1.03 to 1.59, I^2^ = 60%) and reduced length of stay (MD 0.28 days, 95%CI 0.03 to 0.54, I^2^ = 0%) in an acute hospital setting. There was preliminary evidence from one high quality randomised controlled trial that AHA provision of nutritional supplements and assistance with feeding reduced the risk of patient mortality after hip fracture (RR 0.41, 95%CI 0.16 to 1.00). In a small number of studies (*n* = 6) there was no significant difference in patient and organisational outcomes when AHA therapy was substituted for therapy delivered by an allied health professional.

**Conclusion:**

We found preliminary evidence to suggest that the use of AHAs to provide additional therapy may be effective for improving some patient and organisational outcomes.

**Review registration:**

CRD42019127449.

## Background

Allied health assistants (AHAs) are support staff who complete clinical and non-clinical tasks under the supervision and delegation of an allied health professional [1]. An AHA’s role is commonly constructed in order to complement the work of the allied health professionals and assist in the delivery of allied health services across a broad range of clinical settings, including community, rehabilitation, acute care, aged care and mental health [2]. Unlike allied health professionals, AHAs are not university-trained and their qualifications can vary from informal ‘on the job’ training to certificate level qualification [3, 4].

The term ‘allied health’ commonly describes health professionals other than nursing and medical professionals [2]. Allied health professionals have been grouped based on their primary tasks into two categories: ‘therapy’ and ‘scientific’ [5]. Allied health therapy professions have a core focus on providing therapy to treat impairments and improve function; including the dietetic, occupational therapy, physiotherapy, psychology, podiatry and social work professions [2, 5]. The scientific allied health professions perform a key role in the science of healthcare; including the pharmacy, medical science and nuclear medicine professions [2, 5]. Our review will focus on the delegation of tasks by allied health therapy professions to AHAs.

The tasks performed by AHAs can be categorised as either clinical or non-clinical. Clinical tasks include any direct therapeutic interventions provided to patients such as exercise therapy, speech therapy and nutrition advice [6, 7]. AHAs provide this therapy under the supervision of an allied health professional but under many task delegation structures cannot perform clinical tasks that involve evaluation, diagnosing or assessing patient health conditions [6–8]. Therefore, allied health professionals must perform a comprehensive assessment of the patient and prescribe appropriate therapy prior to delegating the AHA to perform any clinical tasks. In contrast, non-clinical tasks may support patient care but do not involve providing therapy directly to the patient [6, 7]. Non-clinical tasks may include administration duties (e.g. completing paperwork for equipment hire or health service referrals), maintenance of equipment and cleaning the clinical environment [6, 7].

In some healthcare settings advanced AHA roles have been implemented where the AHA can work beyond the skill base normally expected of an AHA [9, 10]. These roles are diverse and challenging to define [10]. However, they generally require additional training that enables the advanced AHA greater scope to practice autonomously, make decisions regarding interventions and screen patients [10]. Our review will focus on the practice of the standard AHA role rather than the advanced AHA role.

Delegation of therapy to AHAs refers to the allied health professional prescribing an appropriate treatment for the patient and directing the AHA to provide this therapy. The AHA can provide delegated therapy in collaboration with the allied health professional, assisting with the delivery of therapy that requires more than one person to deliver (e.g. walking a patient who requires a high level of assistance). Alternatively, they can provide delegated therapy independently, administering therapy that has been prescribed by an allied health professional (e.g. conducting a health professional prescribed exercise program with a patient) [6, 7]. During the latter, the AHA is required to report to the allied health professional and feedback information relating to the patients’ therapy.

Delegation of tasks to AHAs may benefit healthcare organisations, healthcare professionals and patients. Healthcare organisations may benefit from an improved workforce capacity, due to lower salaries paid to AHAs [7], with allied health professionals having greater time available to carry out more complex tasks [8, 11]. Some studies have reported that patients are perceived to receive a higher quality of care due to increased face-to-face therapy time [8, 12]. However, despite these purported benefits the allied health professions continue to spend considerable time completing tasks that could be delegated to an AHA [3, 7]. Withholding delegation of clinical tasks to AHAs has been attributed to a number of factors. These include the lack of clarity about AHA scope of practice [3, 13], limited availability to train AHAs in the skills required to deliver therapy [3], and an unwillingness to delegate clinical tasks [3].

The effect of delegation of clinical tasks to AHAs on patient and healthcare organisational outcomes is unknown and may contribute to the unwillingness of the allied health professions to delegate clinical tasks [6]. It is important that the allied health workforce can better delegate to AHAs, as they face the challenge of an increase in demand for health services from an ageing population with increasing complexity of healthcare needs [14, 15]. A better understanding of the effects of the delegation of therapy to AHAs may guide the allied health professions on how to best use the AHA workforce and increase delegation of clinical tasks to AHAs.

The primary aim of this review was to investigate the effect of delegation of therapy to AHAs on adult and paediatric patient outcomes of impairment, activity limitation, participation restriction, safety (including harms of therapy), and satisfaction in hospital and outpatient community settings compared to patients who received less AHA therapy, or therapy from an allied health professional. The secondary aim was to investigate the effect of delegation of therapy to AHAs on organisational outcomes including length of stay, hospital readmission, and cost effectiveness.

## Methods

### Protocol and registration

This systematic review was reported with reference to the Preferred Reporting Items for Systematic Reviews and Meta-analyses (PRISMA) guidelines for high-quality reporting of systematic reviews and meta-analyses [16] and was prospectively registered in the PROSPERO database (registration number: CRD42019127449).

### Eligibility criteria

To be eligible for inclusion studies met the following criteria: (1) participant - included adult or paediatric patients receiving healthcare in a hospital or community outpatient setting; (2) intervention - investigated delegation of therapy to an AHA by a therapy allied health professional [5]; 3) comparator - included a comparator group of patients who did not receive AHA therapy, received less AHA therapy or therapy from only an allied health professional; (4) outcomes - measured patient outcomes (e.g. measures of impairment, activity limitation, participation restriction, safety (including harms of therapy), or satisfaction) or organisational outcomes (e.g. length of stay, hospital readmission, cost effectiveness); (5) research design - used a randomised or non-randomised (e.g. pre-post study design) trial design; (6) written in English language; (7) peer reviewed; (8) written in full text.

For the purpose of this present review studies meeting inclusion criteria were required to investigate delegation by a ‘therapy’ allied health professional and studies investigating delegation of tasks by a ‘science’ allied health professional were excluded [5]. Studies were also excluded if they investigated an expanded scope of practice role where AHAs performed screening tasks in addition to providing therapy [10].

### Information sources

From earliest date available until 18th July 2019, the electronic databases Databases MEDLINE (Ovid), Embase (Ovid), Informit (all databases), Emcare (Ovid), PsycINFO (Ovid), Cumulative Index to Nursing and Allied Health Literature [CINAHL] (EbscoHost) and the Cochrane Database of Systematic Reviews were searched. Citation tracking on Google Scholar and manual searching of the reference lists of included articles and published trial protocols were conducted to ensure all relevant studies were located.

### Search

The concepts of allied health profession and allied health assistant were combined with the ‘AND’ operator. Synonyms and MeSH subject headings were searched for each concept and combined with the ‘OR’ operator. Subject headings were searched for all databases with this option (e.g. Medline, Embase, CINAHL). Searches were conducted on all available fields and not restricted by publication date but were limited to English language.

An example search strategy is provided in an additional file (see Additional file 1). The search strategy reported deviates from our planned search strategy in the registered PROSPERO protocol (CRD42019127449), with the terms ‘allied health’ and ‘nutritionist’ added to the planned search strategy.

### Study selection

Two reviewers (DS, BS) independently screened the articles by title and abstract using the pre-determined eligibility criteria. Any articles that did not meet the criteria were excluded. Full text copies of articles that were not definitely excluded on title and abstract were retrieved for detailed examination. The two reviewers then independently reapplied the eligibility criteria with discussion ensuing to reach a consensus. Where consensus between the two reviewers could not be met, a third reviewer was consulted (CW). Agreement between the two reviewers was reported with the kappa statistic (κ).

### Data collection process

Two authors (DS, BS) used pre-designed spreadsheets to extract data on participants, healthcare interventions and settings, allied health professions who prescribed the therapy/intervention, allied health assistants and outcomes. The spreadsheets were piloted using articles obtained during the literature scope prior to the systematic search.

### Risk of bias in individual studies

All studies were critically appraised for methodological quality by two reviewers (DS, BS) independently using 13 internal validity items from the Downs and Black checklist [17]. The checklist has substantial inter-rater reliability [18] and has been highlighted for use in assessing the quality of non-randomised controlled studies [19, 20]. Any disagreements between reviewers were resolved through discussion. Where consensus could not be reached, a third reviewer was consulted (AD). Inter-rater agreement was reported with the kappa statistic (κ).

### Synthesis of results

Studies were grouped into two categories: (1) studies that investigated the addition of AHA therapy to usual care; and (2) studies that investigated the effect of substituting allied health professional therapy with AHA therapy.

Data analysis was conducted using Review Manager software [21]. Mean differences (MD), standardised mean differences (SMD) and risk ratios (RR) of events were calculated from objective data. Post-intervention mean and standard deviations were used to calculate MD and SMD. Because it is necessary to use mean and standard deviation values to conduct meta-analyses, medians and interquartile ranges were transformed using recommended methods [22]. Meta-analyses were conducted using the inverse variance method and random-effects model where the intervention, allied health professional delegating the intervention, patient population and outcome were similar. If combining data were not appropriate, the reporting of results was provided in a table with a descriptive synthesis. The number needed to treat (NNT) was calculated for statistically significant RR results to help evaluate clinical significance. Statistically significant MDs were compared to established minimal clinical significant difference (MCID) values to determine clinical significance for patient outcomes. MCID values are provided in an additional file (see Additional file 2). The strength of the SMD was determined according to Cohen where 0.2 is considered to be a small effect; 0.5 a moderate effect; and 0.8 a large effect [23]. A moderate effect (0.5) or greater was considered to likely be clinically significant [24].

### Risk of bias across studies

The Grades of Recommendation, Assessment, Development and Evaluation (GRADE) approach was applied to each meta-analysis to determine the quality of evidence [25]. This approach involved downgrading evidence from high to very low quality based on criteria. Downgrading the evidence one place (e.g. high to moderate) occurred if: (1) the majority of studies had at least 4 items on the internal validity scale of the Downs and Black checklist that were not met [17]; (2) substantial statistical heterogeneity existed between studies (I^2^ ≥ 25%) [26]; (3) there was imprecision in the result (i.e. large confidence interval); and (4) the majority of studies in the meta-analysis did not use a randomised controlled trial design.

## Results

### Study selection

**Fig. 1:**
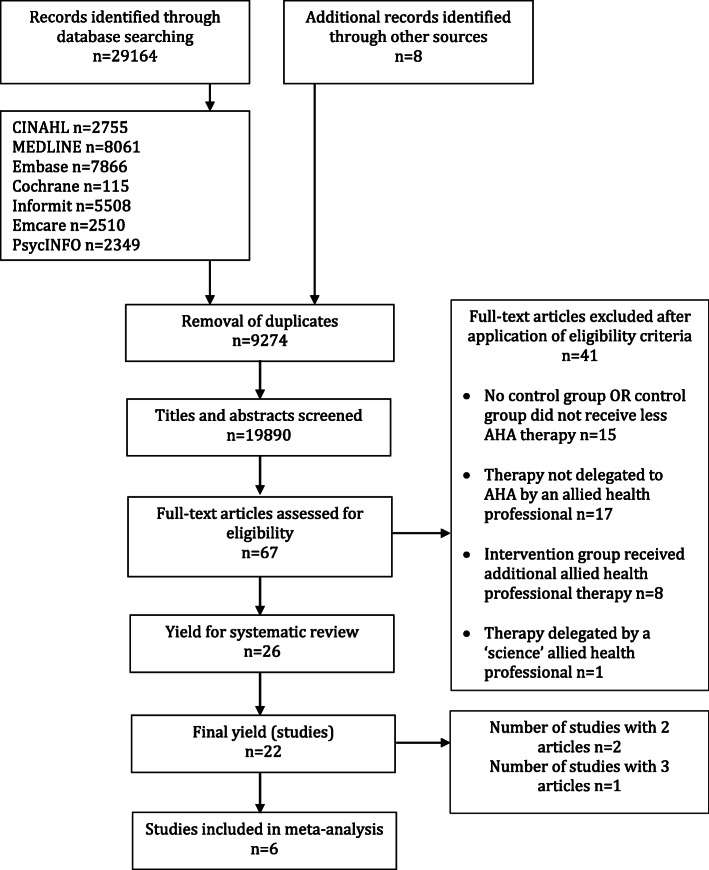
Flow of studies through the review

The database search yielded 29,164 records and an additional 8 records were identified through citation tracking and manual searching of reference lists. Following removal of duplicates 19,890 titles and abstracts were screened. Sixty-seven articles were retrieved for full text review following application of the eligibility criteria to title and abstract. Twenty-six articles fulfilled the inclusion criteria. The twenty-six articles included in the review reported on 22 studies (Fig. 1). Agreement between reviewers was good (κ = 0.79, 95%CI 0.64 to 0.94).

### Study characteristics and risk of bias within studies

Seventeen studies [27–45] investigated the addition of AHA therapy to usual care and six studies [33, 34, 46–52] substitution of allied health professional therapy with AHA therapy with one study [33, 34] investigating both addition and substitution. Ten studies were conducted in the United Kingdom [27, 28, 30, 33, 34, 37, 39, 40, 43–48], seven in Australia [31, 32, 36, 41, 49, 50, 52], two in New Zealand [38, 51], two in the United States of America [29, 42] and one in Finland [35]. A table outlining study characteristics is provided in an additional file (see Additional file 3).

Seventeen studies investigated the addition of AHA therapy to usual care. Of these 17 studies, four studies reported that the AHAs providing the therapy had formal qualifications (certification) [31, 35, 38, 42]. There were five studies where the AHAs were provided with informal training from the allied health professionals for the purposes of the study [28, 33, 34, 37, 40, 44, 45], and training provided or qualifications were not stated in eight studies [27, 29, 30, 32, 36, 39, 41, 43]. Therapy delegated to AHAs included the supervision of the patient performing mobility/lower limb exercise (*n* = 12) [27, 29 30, 32, 35, 36, 38–40, 42–45], upper limb exercise (*n* = 1) [33, 34], cognitive behavioural therapy (*n* = 1) [37], retraining in activities of daily living (ADL) (*n* = 2) [38, 41], provision of nutritional supplements/assistance with feeding (*n* = 2) [28, 40] and assistance transitioning home following hospital admission (*n* = 1) [31]. The details of the AHA interventions are provided in an additional file (see Additional file 4). Therapy was provided to children with cerebral palsy (*n* = 1) [43], adults with stroke (*n* = 3) [27, 30, 33, 34], hip fracture (*n* = 1) [28], fear of falling (*n* = 1) [37], general medical illness (*n* = 8) [29, 31, 32, 35, 36, 38, 41, 42], post-elective lower limb arthroplasty (*n* = 1) [39] and post-ICU admission (*n* = 2) [40, 44, 45]. The majority of studies were conducted in a hospital setting with 10 in an acute setting [28–30, 32, 36, 39–42, 44, 45], one in a sub-acute setting [27] and one in a combination of acute and sub-acute [33, 34]. The remaining five studies were conducted in a community outpatient or home-based setting [31, 35, 37, 38, 43]. Given the nature of the interventions no study blinded participants to group allocation. Failure to conceal group allocation of participants [27, 30, 40, 42, 43] was the predominant internal validity limitation of randomised controlled trials. In addition to not randomising participants, the majority of cohort studies did not adjust for different lengths of follow-up [29, 31, 36, 39, 41], confounders [29, 30, 35, 39, 41], or loss to follow-up in their analyses [31, 36, 39, 41].

Six studies investigated the substitution of allied health professional therapy with AHA therapy. These consisted of three investigating substitution of therapy provided by a physiotherapist [33, 34, 49, 51], one by an occupational therapist [50] and two by a speech pathologist [46–48, 52]. In one study the AHAs providing therapy had formal qualifications [50], in two studies they were provided with informal training from the relevant allied health professional [33, 34, 46–48]. The training or qualifications were not stated in three studies [49, 51, 52]. Therapy provided by AHAs included speech and language therapy (*n* = 2) [46–48, 52], group ADL training (*n* = 1) [50], group functional retraining exercise program (*n* = 1) [49], upper limb exercise (*n* = 1) [33, 34] and community walking program (*n* = 1) [51]. The details of the AHA and allied health professional interventions are provided in an additional file (see Additional file 5). Therapy was provided to children with receptive or expressive language impairment (*n* = 1) [46–48] and adults with neurological aphasia (*n* = 1) [52], stroke (*n* = 3) [33, 34, 49, 51] and neurological or orthopaedic conditions requiring inpatient rehabilitation (*n* = 1) [50]. Studies were conducted in community outpatient (*n* = 2) [46–48, 51], sub-acute (*n* = 2) [49, 50], a combination of acute and sub-acute (*n* = 1) [33, 34] and a combination of sub-acute and community outpatient settings (*n* = 1) [52]. Given the nature of the interventions no trial blinded participants to group allocation. Failure to adjust for loss to follow-up in analyses [33, 34, 50–52] was the predominant methodological risk of bias.

Downs and Black checklist internal validity scale items are reported in an additional file (see Additional file 6). Agreement between reviewers using the Downs and Black tool internal validity items was good (κ = 0.66, 95%CI 0.56 to 0.75).

### Synthesis of results

#### Effect of the addition of AHA therapy to usual care

##### Impairment outcomes

Additional AHA supervised exercise within hospitals had mostly no impact on patient impairments (Table 1) [27, 33, 34, 40, 44, 45]. One study investigated people who had a stroke and measured total body weight-bearing between the affected and non-affected foot during sit to stand transfers. It determined the AHA supervised exercises had a large effect on weight bearing through the affected foot (SMD 1.13, 95%CI 0.11 to 2.14) [27]. The remaining studies found no effect for measures of strength [33, 34, 40, 44, 45], pain [40, 44, 45], depression [44, 45], anxiety [44, 45], or fatigue [40, 44, 45].

**Table 1:** Effect of additional AHA on patient impairment outcomes

Study	Outcome	MD (95%CI)	SMD (95%CI)
Britton 2008 [27]	**Weight through affected foot (% weight)**	**9.10 (2.00 to 16.20)**	**1.13 (0.11 to 2.14)**
Duncan 2006 [28]	**Arm circumference (cm)**	**0.39 (N/A)**^**a**^	N/A
	Triceps skin thickness (mm)	0.34 (N/A) ^**a**^	N/A
	Weight (Kg)	0.65 (N/A) ^**a**^	N/A
	Hand grip strength (Nm)	1.99 (N/A) ^**a**^	N/A
	**Energy intake (Kcal)**	**349 (259 to 439)**	**0.91 (0.67 to 1.16)**
Lincoln 1999 [33]	Hand grip strength (% max unaffected hand)	− 0.67 (− 12.10 to 10.75)^b^	− 0.02 (− 0.47 to 0.44)^b^
Niemela 2012 [35]	Female		
	Hand grip strength (Kg)	− 1.7 (− 4.21 to 0.81)	− 0.20 (− 0.50 to 0.10)
	Knee extension strength (N)	−8.4 (− 48.24 to 31.44)	− 0.06 (− 0.36 to 0.24)
	**Pain VAS (cm)**	**−1.0 (− 1.85 to − 0.15)**	**− 0.35 (− 0.65 to − 0.05)**
	GDS (units)	− 0.50 (− 1.43 to 0.43)	−0.16 (− 0.44 to 0.11)
	Male		
	Hand grip strength (Kg)	−0.80 (− 3.55 to 1.95)	− 0.08 (− 0.35 to 0.19)
	Knee extension strength (N)	− 23.0 (− 61.33 to 15.33)	−0.16 (− 0.44 to 0.11)
	**Pain VAS (cm)**	**0.70 (0 to 1.4)**	**0.27 (0 to 0.54)**
	GDS (units)	−0.40 (− 1.25 to 0.45)	− 0.13 (− 0.40 to 0.14)
Parry 2016 [37]	**FES-I (units)**	**4.02 (2.10 to 5.95)**^**a**^	N/A
	**HADS: depression (units)**	**0.97 (0.33 to 1.62)**^**a**^	N/A
	HADS: anxiety (units)	0.70 (− 0.03 to 1.42) ^**a**^	N/A
Salisbury 2010 [40]	Fatigue VAS (cm)	−0.60 (−5.29 to 4.09)^b^	− 0.14 (− 1.39 to 1.10)^b^
	Pain VAS (cm)	0.96 (−5.77 to 7.69)^b^	0.16 (− 1.08 to 1.40)^b^
	Hand grip strength (Kg)	1.80 (−43.55 to 47.15)^b^	0.06 (− 1.21 to 1.32)^b^
	Calorie intake (% of requirements)	23.24 (− 27.46 to 73.94)^b^	0.53 (−0.83 to 1.88)^b^
	Protein intake (% of requirements)	21.54 (− 36.67 to 79.75)^b^	0.41 (−0.93 to 1.75)^b^
Walsh 2015 [44]	Hand grip strength (Kg)	−1.63 (−4.54 to 1.28)^b^	−0.16 (− 0.45 to 0.13)^b^
	HADS: depression (units)	0.33 (−0.88 to 1.54)^b^	0.08 (− 0.21 to 0.37)^b^
	HADS: anxiety (units)	−0.67 (− 2.30 to 0.96)^b^	− 0.12 (− 0.41 to 0.17)^b^
	Fatigue VAS (cm)	− 0.03 (− 0.97 to 0.91)^b^	−0.01 (− 0.32 to 0.30)^b^
	Pain VAS (cm)	0.23 (−0.74 to 1.20)^b^	0.07 (− 0.24 to 0.38)^b^
	Davidson Trauma Scale (units)	−2.67 (−8.74 to 3.40)^b^	−0.13 (− 0.44 to 0.18)^b^
Weindling 2007 [43]	GMDS (units)	3.2 (− 56.23 to 62.63)	0.03 (− 0.51 to 0.57)

*AHA* Allied health assistant, *FES-I* Fears efficacy scale-international, *GMDS* Griffiths mental development scale, *HADS* Hospital anxiety and depression scale, *MD* Mean difference, *N/A* Not available or unable to be calculated, *SMD* Standardised mean difference, *VAS* Visual analogue scale. Bold text indicates statistically significant difference between groups favouring allied health assistant group

Positive MD favours additional allied health assistant group

^**a**^MD reported in study

^b^MD calculated from converted medians

Additional community AHA supervised exercise had no effect on impairments in a paediatric population with cerebral palsy [43] and a clinically insignificant effect on strength and pain in an elderly population [35, 53].

A study investigating the effect of additional AHA provision of nutritional supplements and assistance with feeding found a large effect on energy intake over a 24-h period (SMD 0.91, 95%CI 0.67 to 1.16) indicative of improved nutrition [28].

One study investigated the effect of additional AHA cognitive behavioural therapy delegated by a psychologist in people with a fear of falling and found significant improvements in fear of falling and depression [37]. The improvement on the Hospital Anxiety and Depression Scale (HADS) depression subscale (MD 0.97 units, 95%CI 0.33 to 1.62) exceeded lower estimates of the MCID (MCID range: 0.5 to 5.57) [54–56].

##### Activity limitation outcomes

Additional AHA therapy during in-hospital care had little effect on activity limitation (Table 2).

**Table 2:** Effect of additional AHA on activity limitation outcomes

Study	Outcome	MD (95%CI)	SMD (95%CI)
Britton 2008 [27]	Time to stand (sec)	0.1 (−0.25 to 0.45)	0.25 (− 0.68 to 1.18)
	Number of sit-to-stands in 1-min	3.00 (0.94 to 6.94)	0.76 (− 0.20 to 1.72)
Howe 2006 [30]	Time to stand (sec)	−0.70 (− 2.81 to 1.41)	−0.25 (− 0.96 to 0.47)
	Time to sit (sec)	0.20 (−0.96 to 1.36)	0.12 (− 0.60 to 0.85)
Isbel 2014 [31]	Lawton ADL scale (units)	−3.48 (− 8.85 to 1.89)	−0.51 (− 1.28 to 0.25)
	Barthel index (units)	−0.35 (− 4.04 to 3.33)	−0.07 (− 0.83 to 0.67)
Jones 2006 [32]	**TUGT (sec) (change score)**	**4.74 (1.54 to 7.94)**^**b**^	**0.64 (0.11 to 1.16)**^**b**^
	Barthel Index (units) (change score)	1.00 (−4.17 to 6.17)^**b**^	0.07 (− 0.28 to 0.42)^**b**^
Lincoln 1999 [33]	Rivermead Arm (units)	0 (− 1.70 to 1.70)^**b**^	0 (−0.30 to 0.30)^**b**^
	ARAT (units)	1.00 (−8.43 to 10.43)^**b**^	0.03 (−0.27 to 0.33)^**b**^
	Barthel Index (units)	−0.33 (− 2.59 to 1.93)^**b**^	− 0.04 (− 0.34 to 0.26)^**b**^
	Rivermead gross motor (units)	−1.00 (− 2.58 to 0.58)^**b**^	−0.19 (− 0.49 to 0.11)^**b**^
	Ten hole peg test (units)	−3.67 (− 15.07 to 17.73)^**b**^	− 0.09 (− 0.39 to 0.20)^**b**^
Parry 1999 [34]	**Rivermead Arm (units)**	**2.00 (0.66 to 3.34)**^**b**^	**0.71 (0.20 to 1.22)**^**b**^
	**ARAT (units)**	**11.66 (2.42 to 20.90)**^**b**^	**0.59 (0.08 to 1.10)**^**b**^
	Barthel Index (units)	2.00 (− 0.51 to 4.51)^**b**^	0.37 (− 0.13 to 0.87)^**b**^
Niemela 2012 [35]	Female		
	Sit to stand 5 times (sec)	−0.60 (− 4.50 to 3.30)	−0.05 (− 0.35 to 0.25)
	Walking speed (m/sec)	0 (−0.14 to 0.14)	0 (− 0.30 to 0.30)
	Berg balance scale (units)	0.50 (−4.75 to 5.75)	0.29 (−0.27 to 0.33)
	Male		
	Sit to stand 5 times (sec)	1.7 (−2.21 to 5.61)	0.12 (−0.15 to 0.39)
	Walking speed (m/sec)	0 (−0.15 to 0.15)	0 (−0.27 to 0.27)
	**Berg balance scale (units)**	**−6.2 (− 10.87 to − 1.53)**	**− 0.36 (− 0.64 to − 0.09)**
Nolan 2008 [36]	Elderly mobility scale (units)	2.46 (N/A)^**a**^	N/A
Parry 2016 [37]	SPPB (units)	0.90 (− 1.06 to 2.87)^**a**^	N/A
	Functional reach test (units)	0.91 (− 0.85 to 2.66)^**a**^	N/A
Parsons 2018 [38]	Inter RAI-CA (units)		
	**bathing**	**0.15 (N/A)**^**a**^	N/A
	**dressing lower body**	**0.10 (N/A)**^**a**^	N/A
	Hygiene	0.09 (N/A)^**a**^	N/A
	Locomotion	0.08 (N/A)^**a**^	N/A
	Toilet use	0.03 (N/A)^**a**^	N/A
	Meal preparation	0.08 (N/A)^**a**^	N/A
	House work	− 0.03 (N/A)^**a**^	N/A
	Medication	0.01 (N/A)^**a**^	N/A
	Stairs	−0.03 (N/A)^**a**^	N/A
Pengas 2015 [39]	Hip arthroplasty		
	**Time to mobilise with two sticks (days)**	**0.42 (0.12 to 0.72)**	**0.30 (0.09 to 0.51)**
	Knee arthroplasty		
	**Time to mobilise with two sticks (days)**	**0.58 (0.27 to 0.89)**	**0.41 (0.15 to 0.67)**
Salisbury 2010 [40]	Rivermead mobility index (units)	−1.93 (−11.58 to 7.72)^**b**^	− 0.23 (− 1.42 to 0.97)^**b**^
	TUGT (sec)	−3.46 (− 26.49 to 19.57)^**b**^	−0.19 (− 1.51 to 1.13)^**b**^
	10 m walk test (sec)	−9.43 (−47.73 to 28.87)^**b**^	− 0.33 (− 1.66 to 1.00)^**b**^
	Shuttle walk test (m)	−14.50 (− 375.82 to 346.82)^**b**^	− 0.05 (− 1.36 to 1.27)
Shearer 2013 [41]	Barthel Index (units) (change score)	4.27 (N/A)^**a**^	N/A
Siebens 2000 [42]	**Number of independent ADL**	**0.50 (0 to 1.00)**	**0.24 (0.10 to 0.49)**
	NHIS physical activity scale (units)	0.70 (− 0.58 to 1.98)	0.13 (− 0.11 to 0.37)
Walsh 2015 [44]	Rivermead mobility index (units)	0 (− 0.78 to 0.78)	0 (− 0.26 to 0.26)
	TUGT (secs)	0.06 (− 1.31 to 1.43)	0.01 (− 0.28 to 0.31)
Weindling 2007 [43]	GMFM-66 (units)	4.5 (− 10.93 to 19.93)	0.16 (− 0.38 to 0.70)
	Vineland adaptive behaviour daily living scale (units)	1.0 (− 7.04 to 9.04)	0.07 (− 0.47 to 0.61)

*ADL* Activities of daily living, *AHA* Allied health assistant, *ARAT* Action research arm test; *Inter RAI-CA* Inter RAI contact assessment, *FIM* Functional independence measure, *GMFM-66* Gross motor function measure, *MD* Mean difference, *N/A* Not available or unable to be calculated, *SMD* Standardised mean difference, *NHIS* National health interview survey, *SPPB* Short physical performance battery, *TUGT* Timed up and go test. Bold text indicates statistically significant difference between groups favouring allied health assistant group

Positive MD favours additional allied health assistant group

^a^MD reported in study

^b^MD calculated from converted medians

One study found hospital inpatients who received additional AHA supervised exercise had moderate improvements in timed up and go performance (SMD 0.64, 95%CI 0.11 to 1.16) [32]. A further eight studies found that in-hospital AHA exercise had no or clinically insignificant effects on mobility outcomes [27, 30, 33, 34, 36, 39, 40, 42, 44, 45].

Similarly, additional AHA supervised exercise in the community had no or clinically insignificant effects on mobility and balance outcomes for children and adults living in the community [35, 43, 57].

Additional AHA in-hospital upper limb exercise had no effect on upper limb function in people with varying severity of stroke [33, 34]. However, in people with mild upper limb impairment, additional exercise resulted in significant improvements in upper limb function measured on the Action Research Arm Test (SMD 0.71, 95%CI 0.20 to 1.22) and Rivermead Motor Assessment arm subscale (SMD 0.59, 95%CI 0.08 to 1.10). Improvement in the Action Research Arm Test did not reach the MCID threshold of 12 [58].

Additional AHA ADL re-training and exercise had no or clinically non-significant effect on ADL performance in the hospital and community settings [31, 38, 41, 42].

##### Participation restriction outcomes

**Fig. 2:**
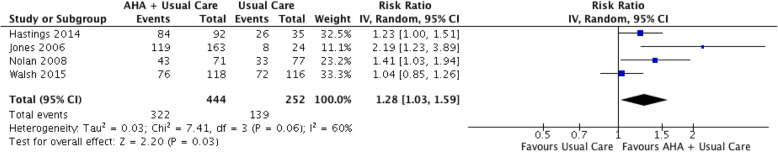
Effect of additional inpatient AHA supervised exercise on discharge to home

Four studies investigated the effect of additional in-hospital exercise on participation restriction [29, 32, 36, 44, 45]. Meta-analysis of these studies with 696 participants provided very low level evidence that additional AHA improved the likelihood of participants discharging to home from acute hospital wards (RR 1.28, 95%CI 1.03 to 1.59, I^2^ = 60%) (Fig. 2) (Table 3). The NNT for a patient to benefit from additional AHA was 6 (95%CI 4 to 10).

**Table 3:** Effect of additional AHA supervised exercise: summary of meta-analyses

Outcome	No. of trials	No. of participants	MD/RR (95%CI), I^**2**^	Quality of evidence (GRADE)
Discharge home	4 [29, 32, 36, 44]	696	RR 1.28 (1.03 to 1.59), I^2^ = 60%	Very Low ^a, b, d^
Acute length of stay (days)	6 [29, 32, 36, 39, 42, 44]	1787	MD − 0.28 (− 0.54 to − 0.03), I^2^ = 0%	Low ^a, d^

*AHA* Allied health assistant, *MD* Mean difference, *RR* Risk ratio, *GRADE* Grading of Recommendations Assessment, Development and Evaluation

Negative MD favours additional AHA intervention group: Acute length of stay

RR > 1 favours additional AHA intervention group: Discharge home

Reason for downgrade: a – risk of bias: majority of trials have at least 4 items on the internal validity scale of the Downs and Black checklist that were not met; b – heterogeneity: I^2^ ≥ 25%; c – imprecision of result: large CI; d - majority of studies not RCT design

One study found that additional AHA in-hospital exercise significantly reduced the number of aged care assessment referrals (RR 0.52, 95%CI 0.18 to 1.44) and approvals (RR 0.46, 95%CI 0.24 to 0.90) [36]. This study was conducted in an Australian healthcare setting where assessments from aged care health professionals are required prior to placement in residential care (Table 4).

**Table 4:** Effect of additional AHA on participation restriction outcomes

Study	Outcome	RR (95%CI)	NNT (95%CI)
Hastings 2014 [29]	**Discharge home**	**1.23 (1.00 to 1.51)**	**6 (3 to 70)**
Jones 2006 [32]	**Discharge home**	**2.19 (1.23 to 3.89)**	**6 (3 to 54)**
Nolan 2008 [36]	**Discharge home**	**1.41 (1.03 to 1.94)**	**3 (2 to 5)**
	**ACAT referral**	**0.40 (0.22 to 0.71)**	**4 (2 to 22)**
	**lACAT approval**	**0.46 (0.24 to 0.90)**	**6 (N/A)**
Shearer 2013 [41]	Discharge home	1.41 (0.76 to 2.63)	–
Walsh 2015 [44]	Discharge home	1.04 (0.85 to 1.59)	–

*ACAT* Aged care assessment team, *AHA* Allied health assistant, *RR* Risk ratio, *N/A* Not available or unable to be calculated, *NNT* Number needed to treat. Bold text indicates statistically significant difference between groups favouring allied health assistant group

Positive MD favours additional allied health assistant group

RR > 1 favours additional allied health assistant group: discharge home

RR < 1 favours additional allied health assistant group: aged care assessment referrals/approvals

^**β**^ MD reported in study

Additional AHA in-hospital ADL re-training and community cognitive behavioural therapy had no effect on discharge home and socialisation/loneliness, respectively [37, 41].

##### Safety outcomes

Six studies investigated the effect of additional AHA therapy on patient safety outcomes [28, 29, 32, 37, 42, 44, 45].

The effect of additional AHA provision of nutritional supplements and assistance with feeding on patient mortality was investigated in one study, which found a significant reduction in mortality in patients post-hip fracture surgery (Table 5) [28].

**Table 5:** Effect of additional AHA on safety outcomes

Study	Outcome	RR (95%CI)	NNT (95%CI)
Duncan 2006 [28]	**Mortality (trauma unit)**	**0.41 (0.16 to 1.00)**	**17 (9 to 315)**
	Mortality (in hospital)	0.56 (0.29 to 1.09)	–
	**Mortality (4-months)**	**0.57 (0.34 to 0.95)**	**11 (5 to 79)**
Hastings 2014 [29]	Proportion of fallers	0.38 (0.02 to 5.92)	–
	Mortality (1-month post D/C)	0.76 (0.15 to 3.97)	–
Jones 2006 [32]	Proportion of fallers	2.17 (0.41 to 11.48)	–
	Medical status deterioration	4.34 (0.50 to 37.90)	–
	Mortality (in hospital)	2.17 (0.41 to 11.48)	–
Parry 2016 [37]	Proportion of fallers	0.96 (0.78 to 1.17)	–
	Adverse events	0.76 (0.44 to 1.30)	–
Siebens 2000 [42]	Mortality (in hospital)	5.07 (0.25 to 104.66)	–
	Mortality (1-month post D/C)	1.02 (0.44 to 2.38)	–
Walsh 2015 [44]	Mortality (in hospital)	0.93 (0.31 to 2.80)	–

*AHA* Allied health assistant, *D/C* Discharge, *RR* Risk ratio, *N/A* Not available or unable to be calculated, *NNT* Number needed to treat. Bold text indicates statistically significant difference between groups favouring allied health assistant group

RR < 1 favours additional allied health assistant group

Additional AHA in-hospital exercise therapy had no effect on patient safety outcomes including mortality [29, 32, 42, 44, 45], mortality 1-month post-discharge from hospital [29, 32], falls [29, 32] or deterioration of medical status [32].

##### Other outcomes

Additional AHA therapy had no effect on other outcomes such as quality of life and patient satisfaction (Table 6).

**Table 6:** Effect of additional AHA on other outcomes

Study	Outcome	MD (95%CI)	SMD (95%CI)
Duncan 2006 [28]	Patient satisfaction nutritional care (units)	N/A	N/A
Niemela 2012 [35]	Female Leipad HRQOL questionnaire (units)	2.50 (− 0.95 to 5.95)	0.22 (− 0.08 to 0.52)
	Male Leipad HRQOL questionnaire (units)	0.70 (− 2.61 to 4.01)	0.06 (− 0.21 to 0.33)
Parry 2016 [37]	EQ-5D 5 L (units)	0.01 (− 0.04 to 0.05)^**a**^	N/A
	WHOQOL (units)	0.85 (− 1.56 to 3.26)^**a**^	N/A
	SF-36 PCS (units)	0.99 (− 0.91 to 2.90)^**a**^	N/A
	SF-36 MCS (units)	1.17 (−1.51 to 3.84)^**a**^	N/A
Walsh 2015 [44]	Patient satisfaction physiotherapy (units)	1.20 (− 0.60 to 3.00)^b^	0.20 (− 0.10 to 0.49)^b^
	SF-12 PCS (units)	− 0.33 (− 4.11 to 3.45)^b^	− 0.02 (− 0.30 to 0.26)^b^
	SF-12 MCS (units)	−1.00 (− 5.54 to 3.54)^b^	− 0.06 (− 0.34 to 0.22)^b^

*AHA* Allied health assistant, *EQ-5D* Euroqol 5 dimension health outcome questionnaire, *HRQOL* Health related quality of life, *MD* Mean difference, *N/A* Not available or unable to be calculated, *SMD* Standardised mean difference, *SF-12 MCS* 12-item short form survey mental component score, *SF-12 PCS* 12-item short form survey physical component score, *SF 36 MCS* 36-item short form survey mental component score, *SF-36 PCS* 36-item short form survey physical component score, *WHOQOL* World Health Organisation quality of life questionnaire. Bold text indicates statistically significant difference between groups favouring allied health assistant group

Positive MD favours additional allied health assistant group

^a^MD reported in study

^b^MD calculated from converted medians

##### Organisational outcomes

**Fig. 3:**
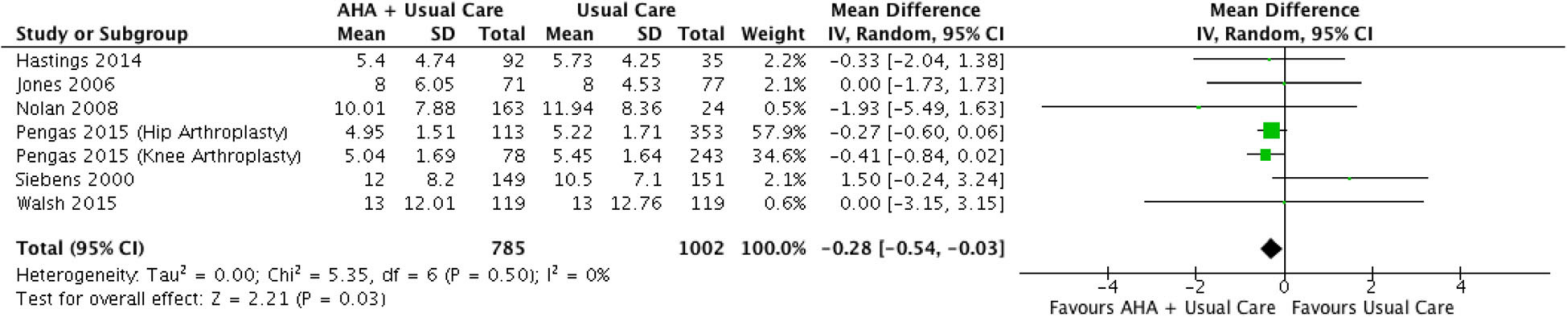
Effect of additional inpatient AHA supervised exercise on acute hospital length of stay (days)

Meta-analysis of six studies [29, 32, 36, 39, 42, 44, 45] with 1787 participants provided low quality evidence that additional exercise supervised by AHAs in an acute hospital setting reduced length of stay by 0.28 days (95%CI 0.03 to 0.54, I^2^ = 0%) (Fig. 3) (Table 3).

One study conducted in a community outpatient setting and not included in the meta-analysis found that additional home-based AHA exercise combined with ADL re-training significantly reduced hospital length of stay by 5.90 days (95%CI 0.55 to 11.25) (Table 7) [38].

**Table 7:** Effect of additional AHA on organisational outcomes

Study	Outcome	MD (95%CI)	RR (95%CI)
Duncan 2006 [28]	Acute hospital LOS (days)	N/A	–
	Acute + sub-acute hospital LOS (days)	N/A	
Hastings 2014 [29]	Acute hospital LOS (days)	− 0.33 (− 2.04 to 1.38)	–
	30-day readmission	–	1.22 (0.48 to 3.07)
	30-day emergency visit	–	1.14 (0.53 to 2.44)
Jones 2006 [32]	Acute hospital LOS (days)	0 (− 1.73 to 1.73)^a^	–
	Acute + sub-acute hospital LOS (days)	−3.00 (− 6.30 to 0.30)^a^	–
Nolan 2008 [36]	Acute hospital LOS (days)	− 1.93 (− 5.49 to 1.63)	–
	28-day readmission	–	0.52 (0.18 to 1.44)
Parsons 2018 [38]	**Hospital LOS prior to commencement of home AHA program (days)**	−**5.90 (− 11.25 to − 0.55)**^a^	–
	Hospital LOS during re-admission in 6-months post commencement of program (days)	− 5.40 (− 11.11 to 0.31)	–
Pengas 2015 [39]	Acute hospital LOS (post elective hip arthroplasty) (days)	− 0.27 (− 0.60 to 0.06)	–
	Acute hospital LOS (post elective knee arthroplasty) (days)	− 0.41 (− 0.84 to 0.02)	–
Shearer 2013 [41]	Acute hospital LOS (days)	N/A	–
Siebens 2000 [42]	Acute Hospital LOS (days)	1.50 (− 0.24 to 3.24)	–
Walsh 2015 [44]	Acute Hospital LOS (days)	0 (−3.15 to 3.15)^a^	–

*AHA* Allied health assistant, *LOS* Length of stay, *MD* Mean difference, *N/A* Not available or unable to be calculated, *RR* Risk ratio

Negative MD favours additional allied health assistant group

RR less than 1 favours additional allied health assistant group

Bold text* indicates statistically significant difference between groups favouring allied health assistant group

^a^MD calculated from converted medians

Three studies found that additional AHA had limited cost effectiveness [37, 43–45]. Cost effectiveness analysis of additional AHA exercise for children with cerebral palsy showed that the incremental healthcare cost per unit improvement in gross motor function measure was £22.39 [43]. Cost effectiveness analysis of additional AHA cognitive behavioural therapy for people with fear of falling showed that the incremental cost per reduction in FES-I unit was £71 and £24 from a societal and healthcare perspective respectively [37].

#### The effect of substituting allied health professional therapy with AHA therapy

##### Patient outcomes

There were no differences between substitution model groups for impairment, activity limitation, participation restriction or other patient outcomes within six studies with varied populations and outcomes (Table 8) [33, 34, 46–52].

**Table 8:** Effect of substitution of allied health professional therapy with AHA therapy on patient outcomes

Study	Outcome	MD (95%CI)	SMD (95%CI)
**Impairment**			
Boyle 2007 [46]	Individual		
	CELF-R (units)	−1.76 (− 6.43 to 2.91)	− 0.18 (− 0.66 to 0.30)
	CELF- E (units)	− 1.35 (− 5.28 to 2.58)	− 0.17 (− 0.65 to 0.31)
	Group		
	CELF-R (units)	−1.33 (− 6.19 to 3.53)	− 0.14 (− 0.63 to 0.35)
	CELF-E (units)	0.47 (− 3.11 to 4.05)	0.07 (− 0.42 to 0.56)
Cox 2014 [50]	AusTOMs impairment: UL (units)	0.45 (− 0.03 to 0.93)	0.44 (− 0.03 to 0.93)
	AusTOMs impairment: Daily tasks (units)	0.22 (− 0.19 to 0.63)	0.25 (− 0.21 to 0.73)
	AusTOMs impairment: Domestic life (units)	0.27 (−0.12 to 0.66)	0.32 (− 0.15 to 0.80)
Lincoln 1999 [33]	Handgrip strength (% max unaffected hand)	8 (−2.06 to 18.06)	0.24 (− 0.06 to 0.54)
**Activity limitation**			
Cannel 2018 [49]	Functional reach (cm) (change score)	0.9 (−3.1 to 5.0)^**a**^	N/A
	Lateral reach (cm) (change score)	2.4 (−0.9 to 5.7)^**a**^	N/A
	Sitting balance (units) (change score)	0.2 (−0.17 to 0.6)^**a**^	N/A
	MMAS-upper arm (units) (change score)	−0.1 (− 0.17 to 0.6)^**a**^	N/A
	Box and block test (units) (change score)	−2.2 (− 7.8 to 3.0)^**a**^	N/A
	Step test (number) (change score)	−0.5 (−2.3 to 1.4)^**a**^	N/A
	TUGT (sec) (change score)	4.9 (− 5.3 to 15.4)^**a**^	N/A
	Walking speed (m/sec) (change score)	0.05 (−0.09 to 0.19)^**a**^	N/A
Cox 2014 [50]	AusTOMs activity limitation: UL (units)	0.35 (−0.09 to 0.79)	0.37 (− 0.10 to 0.85)
	AusTOMs activity limitation: Daily tasks (units)	−0.18 (− 0.51 to 0.15)	−0.26 (− 0.74 to 0.21)
	AusTOMs domestic life: Domestic life (units)	0 (−0.38 to 0.38)	0 (− 0.47 to 0.47)
	FIM (units)	−1.76 (− 6.43 to 2.91)	− 0.18 (− 0.66 to 0.30)
Lincoln 1999 [33]	Rivermead arm (units)	0.66 (− 1.16 to 2.48)^b^	0.11 (− 0.19 to 0.41)^b^
	ARAT (units)	3.33 (− 5.88 to 12.54)^b^	0.11 (− 0.19 to 0.41)^b^
	Barthel index (units)	0 (−2.07 to 2.07)^b^	0 (− 0.30 to 0.30)^b^
	Rivermead gross motor (units)	0 (−1.49 to 1.49)^b^	0 (− 0.30 to 0.30)^b^
	Ten hole peg test (units)	6.33 (−2.10 to 14.76)^b^	0.23 (−0.07 to 0.53)^b^
Parry 1999 [34]	Rivermead arm (units)	0.66 (− 0.45 to 1.77)^b^	0.33 (− 0.22 to 0.88)^b^
	**ARAT (units)**	**7.66 (0.61 to 14.71)**^b^	**0.57 (0.01 to 1.13)**^b^
	Barthel Index (units)	3.00 (0.36 to 5.64)^b^	0.63 (0.07 to 1.19)^b^
**Activity limitation**			
Lord 2008 [51]	Walking speed (m/min)	−2.6 (− 15.2 to 10.0)^**a**^	N/A
	6-min walk test (m)	− 1.1 (− 60.2 to 58)^**a**^	N/A
	ABCS (units)	−0.6 (− 14.8 to 13.5)^**a**^	N/A
Wenke 2014 [52]	CAT spoken language (units)	−3.72 (− 32.84 to 25.40)	−0.14 (− 1.13 to 0.86)
	CAT disability (units)	− 16.87 (− 34.10 to 0.36)	−1.06 (− 2.12 to 0.01)
**Participation restriction**			
Cox 2014 [50]	AusTOMs: Participation restriction (units)	0.15 (− 0.18 to 0.48)	0.21 (− 0.26 to 0.69)
Lord 2008 [51]	SIPSO (units)	0.3 (− 3.9 to 4.5)^**a**^	N/A
**Other**			
Cox 2014 [50]	AusTOMs: Distress / wellbeing (units)	0.03 (− 0.30 to 0.36)	0.04 (− 0.43 to 0.51)

*ABCS* Activities-specific balance confidence scale, *ADL* Activities of daily living, *AHA* Allied health assistant, *ARAT* Action research arm test, *AUS* Australia, *AusTOMs* Australian therapy outcome measures for Occupational Therapy, *CAT* Comprehensive aphasia test, *CELF-E* Clinical evaluation of language fundamentals expressive sub-scale, *CELF-R* Clinical evaluation of language fundamentals receptive sub-scale, *FIM* Functional independent measure, *MD* Mean difference, *MMAS* Modified motor assessment scale, *N/A* Not available or unable to be calculated, *SMD* Standardised mean difference SIPSO: subjective index of physical and social outcome. Bold text* indicates statistically significant difference between groups favouring allied health assistant group

Positive MD favours allied health assistant group

^**a**^MD reported in study

^b^MD calculated from converted medians

One study was conducted in an acute/inpatient rehabilitation setting and reported clinically non-significant improvements on the Action Research Arm Test (MD 7.66 units, 95%CI 0.61 to 14.71, MCID 12) for people with mild upper limb who received AHA therapy [33, 34, 58].

Two studies reported patient safety outcomes [49, 51]. Both these studies reported that there were no adverse events during either AHA or allied health professional led therapy [49, 51].

##### Organisational outcomes

Cost effectiveness analysis showed that there was an additional healthcare cost of £9 per unit of improvement on the clinical evaluation of language fundamentals measure for allied health professional therapy compared to AHA therapy for children with language impairment [46–48].

## Discussion

This review found low level evidence that additional AHA exercise therapy may lead to clinically significant improvement in rates of patients who discharge home, with an additional person discharged home for every six that received additional AHA therapy. This review also found low level evidence that additional AHA exercise therapy may lead to small (0.28 days) reductions in length of stay in an acute hospital setting. There was preliminary evidence, from one high quality randomised controlled trial, that additional AHA provision of nutritional supplements and assistance with feeding may reduce mortality in patients post hip fracture surgery. In a small number of studies there was no evidence to suggest that either allied health professional or AHA therapy was superior.

While more evidence is required to confirm the effects of additional AHA therapy on patient and organisational outcomes, the results of this systematic review are consistent with previous findings investigating the effects of additional therapy. Peiris et al. [59] found that additional physical therapy in the acute and sub-acute hospital settings resulted in small reductions in length of stay and measures of activity limitation and participation. Our findings suggest that providing additional exercise therapy supervised by AHAs may yield similar effects and is an alternative to providing additional exercise supervised by physiotherapists. Similarly, guidelines recommend people with hip fracture receive tailored interventions for improving nutritional intake post-surgery [60]. Our findings suggest that this care can be provided safely and effectively by AHAs and may lead to reduced rates of mortality following hip fracture surgery.

Studies investigated AHA therapy that was prescribed and supervised by allied health professionals and this is an important consideration when implementing AHA therapy. The role of allied health professionals in assessing the patient and prescribing the appropriate therapy to address their needs is crucial. Without this assessment and correct prescription, AHAs could not provide effective therapy. The AHA also requires ongoing supervision, by an allied health professional, to ensure that the therapy is appropriately progressed to meet the patients’ needs (effective care) and does not harm patients if their condition changes (safe care). While it is clear that all therapy was prescribed and supervised in the studies included in this review, the characteristics of the supervision provided were sparsely reported. This is an important consideration for future studies that investigate the effect of AHA therapy on patient outcomes.

This review found that the effect of additional AHA therapy has been mostly investigated in the acute hospital setting where allied health resources for therapy are limited. The role of the allied health professional in this setting is primarily to provide assessment and discharge planning services [41, 60]. As such, there is less focus on intervening to improve patient outcomes (e.g. independence with mobility or ADLs) [40, 61]. In contrast, the AHA clinical role primarily involves providing therapy [7, 32, 40]. Given that previous evaluations on increasing allied health services in the acute setting have shown that its effects on patient and organisational outcomes are unclear, future evaluations on additional allied health services may consider the effects of increasing the ratio of AHA to allied health professional staffing [62, 63].

Some allied health professionals perceive that the therapy they provide may be of more benefit to the patient than AHA therapy [4, 16]. Despite this, there was no evidence, across a small number of studies in both hospital and outpatient settings, to suggest that either type of therapy was superior. The effectiveness of AHA therapy compared to allied health professional therapy remains unclear. It is important that future trials incorporate designs, such as non-inferiority trials, that can investigate the equivalence between AHA and allied health professional therapy. Such trials will help to better understand the effectiveness of AHA therapy and will help guide how the allied health professions use the AHA workforce.

Increasing demands on healthcare systems across the world dictate that investment in the AHA workforce is likely to increase. Our findings provide healthcare organisations and policy makers with an indication of where delegation of therapy to AHAs may be most effective [14, 15]. Specifically, AHAs can impact positively on patient health outcomes when they provide evidence-based therapy, such as nutrition care during post-operative recovery and interventions aimed at increasing physical activity [59, 60]. Encouragingly, of the eight studies included in our review that reported patient safety outcomes none reported an increased risk of harm to patients who received AHA therapy in the hospital or outpatient community settings [28, 29, 32, 37, 42, 44, 49, 51]. Therefore, healthcare organisations should be confident that AHAs can safely provide therapy under the supervision of allied health professionals in a range of healthcare settings and should seek further opportunities for AHAs to provide high quality, evidence-based care.

Limitations to this review include the search strategy, inclusion criteria and risk of bias assessment. The search strategy reported deviates from our planned search strategy in the registered PROSPERO protocol (CRD42019127449). To improve the thoroughness of our search strategy we included the terms ‘allied health’ and ‘nutritionist’. We did not search large multi-disciplinary databases, such as Web of Science and Scopus. However, we did search two of the large multi-disciplinary, medical databases (MEDLINE, Embase) recommended by the Cochrane Collaboration for conducting systematic reviews on healthcare interventions [19], and several databases specific to the allied health professions (CINAHL, Emcare, PsychINFO). Not ‘exploding’ MeSH terms may have led to relevant articles not being identified in our search. However, our database search strategy did include terms for all allied health therapy professions and identified the majority of studies included in this review (*n* = 20) with only 3 included studies identified by hand searching reference lists and citation tracking. Including only peer-reviewed articles (i.e. not searching grey literature) written in English may have also led to relevant studies being missed. There is no consensus on the preferred appraisal tool to assess the risk of bias of non-randomised controlled studies [20]. We chose the Downs and Black checklist as it has substantial inter-rater reliability [18] and has been highlighted for use in assessing the quality of non-randomised controlled studies [19, 20].

The main limitation of the studies included in this review is the low quality of studies with many of the significant findings from this review derived from cohort studies. While there is value in analysing the results from both cohort studies and randomised controlled trials, further high quality trials are warranted to confirm the findings in this systematic review [64]. However, randomised controlled trials are not always the most appropriate design for testing the effectiveness of complex health interventions and can lack external validity, or generalizability, as they are usually conducted under controlled and resource intensive conditions [65, 66]. Cohort studies allow estimations of effects in settings that are more ‘real world’ than randomised trials and can generate valid results [66]. Therefore, the results of cohort studies should not be dismissed when making informed decisions about clinical practice and healthcare resource utilisation [66].

Another limitation is that few studies described the level of AHA supervision that was provided by allied health professionals. Similarly AHA training or qualification was only described in about half of the included studies. This makes it difficult to translate findings into practice as the qualifications required to work as an AHA vary [8, 67]. Generalisability of our findings may be affected by the geographical biases of study locations. The majority of studies were conducted in the UK, Australia and New Zealand, where healthcare systems and the role of allied health share many commonalities. Findings may not be generalisable to healthcare settings in other countries where the role of AHAs and allied health professionals and funding models can differ [2, 6]. Last, the effect on patient outcomes measured in this review is dependent on two factors: the ability of the AHA to deliver therapy; and the effectiveness of the therapy prescribed to patients. It is possible in studies that found no effect on patient outcomes that this is reflective of the effectiveness of the intervention rather than the ability of the AHA. This highlights the importance of investigating the effect of delegating evidence-based interventions to AHAs.

Further research should focus on investigating the effects of delegation of therapy to AHAs on patient and organisational outcomes in a range of healthcare settings including hospital, rehabilitation and community settings. The community setting is of particular importance as demand for community care increases [14]. Researchers should also be encouraged to investigate a range of therapies, delegated by a variety of allied health professions, and the use of AHAs to assist in the implementation of evidence-based practice. The majority of studies have focused on exercise interventions delegated by the physiotherapy profession, with other forms of therapy largely overlooked. Studies investigating the effect of substitution of therapy should consider using a non-inferiority trial design to establish equivalence between AHA and allied health professional therapy. While large randomised controlled trials are required, cohort studies provide valuable information that reflects the real world healthcare setting and therefore, a combination of both study designs is recommended [66]. Last, better reporting on the level of AHA supervision and qualifications/training is required to ensure that research findings can be translated into practice.

## Conclusion

We found preliminary evidence to suggest that the use of AHAs to provide additional therapy may be effective for improving some patient and organisational outcomes. In a small number of studies there was no significant difference in patient and organisational outcomes when AHA therapy was substituted for allied health professional therapy.

## Supplementary information

**Additional file 1.** Example search strategies. Example of the search strategy used to search Ovid Medline, Ovid Embase and Cumulative Index to Nursing and Allied Health Literature (CINAHL) databases

**Additional file 2.** Minimum clinically important difference values. Minimum clinically important difference values used to determine clinical significance of findings

**Additional file 3.** Study characteristics. Characteristics of the included studies

**Additional file 4.** Intervention characteristics: Additional AHA. Details of the additional AHA interventions for each study

**Additional file 5.** Intervention characteristics: Substitution AHA. Details of the substitution AHA interventions for each study

**Additional file 6.** Downs and Black internal validity items. Study compliance with Downs and Black internal validity items

## Data Availability

The datasets used and analysed during the current review are available from the corresponding author on reasonable request.

## Abbreviations

AHA: Allied health assistant
GRADE: Grades of recommendation, assessment, development and evaluation
MCID: Minimum clinically important difference
MD: Mean difference
NNT: Number needed to treat
RR: Risk ratio
SMD: Standardised mean difference
