# Supplementary material for: The effect of delegation of therapy to allied health assistants on patient and organisational outcomes: a systematic review and meta-analysis
Source: BMC Health Serv Res. 2020 Jun 3;20:491. doi: 10.1186/s12913-020-05312-4 (PMC7268306; doi:10.1186/s12913-020-05312-4)
Supplement: Supplementary file 1 — Additional file 1. Example search strategies. Example of the search strategy used to search Ovid Medline, Ovid Embase and Cumulative Index to Nursing and Allied Health Literature (CINAHL) databases [file 12913_2020_5312_MOESM1_ESM.docx]

**Additional file 1**. Example Search Strategies

Ovid Medline search strategy

| 1. | Allied Health Occupations/ or allied health.mp. |
| --- | --- |
| 2. | exercise physiolog*.mp. |
| 3. | Art Therapy/ or art therap*.mp. |
| 4. | Chiropractic/ or chiropract*.mp. |
| 5. | (dietician* or dietitian* or dietetic* or nutritionist*).mp. or Nutritionists/ |
| 6. | physiotherap*.mp. |
| 7. | Physical Therapists/ or physical therap*.mp. |
| 8. | Occupational Therapy/ or occupational therap*.mp. |
| 9. | Social Work/ or social work*.mp. |
| 10. | Music Therapy/ or music therap*.mp. |
| 11. | Psychology/ or psycholog*.mp. |
| 12. | Podiatry/ or podiatr*.mp. |
| 13. | chiropod*.mp. |
| 14. | osteopath*.mp. |
| 15. | orthotist*.mp. |
| 16. | prosthetist*.mp. |
| 17. | speech therap*.mp. |
| 18. | speech patholog*.mp. |
| 19. | speech language therap*.mp. |
| 20. | oral health*.mp. |
| 21. | 1 or 2 or 3 or 4 or 5 or 6 or 7 or 8 or 9 or 10 or 11 or 12 or 13 or 14 or 15 or 16 or 17 or 18 or 19 or 20 |
| 22. | assistant*.mp. |
| 23. | support worker*.mp. |
| 24. | technician*.mp |
| 25. | 22 or 23 or 24 |
| 26. | 21 and 25 |

**.mp**: free text searching in OVID Medline.

Ovid Embase search strategy

| 1. | allied health.mp. |
| --- | --- |
| 2. | exercise physiolog*.mp. |
| 3. | Art Therapy/ or art therap*.mp. |
| 4. | Chiropractic/ or chiropract*.mp. |
| 5. | Dietitian/ or (dietician* or dietitian* or dietetic* or nutritionist*).mp. |
| 6. | Physiotherapist/ physiotherap*.mp. |
| 7. | physical therap*.mp. |
| 8. | Occupational Therapy/ or occupational therap*.mp. |
| 9. | Social Work/ or social work*.mp. |
| 10. | Music Therapy/ or music therap*.mp. |
| 11. | Psychology/ or psycholog*.mp. |
| 12. | Podiatry/ or podiatr*.mp. |
| 13. | chiropod*.mp. |
| 14. | osteopath*.mp. |
| 15. | orthotist*.mp. |
| 16. | prosthetist*.mp. |
| 17. | speech therap*.mp. |
| 18. | speech patholog*.mp. |
| 19. | speech language therap*.mp. |
| 20. | oral health*.mp. |
| 21. | 1 or 2 or 3 or 4 or 5 or 6 or 7 or 8 or 9 or 10 or 11 or 12 or 13 or 14 or 15 or 16 or 17 or 18 or 19 or 20 |
| 22. | assistant*.mp. |
| 23. | support worker*.mp. |
| 24. | technician*.mp |
| 25. | 22 or 23 or 24 |
| 26. | 21 and 25 |

**.mp**: free text searching in OVID Medline.

Cumulative Index to Nursing and Allied Health Literature (CINAHL) search strategy

| 1. | (MH “Allied Health Professions”) OR allied health |
| --- | --- |
| 2. | exercise physiolog* |
| 3. | (MH “Art Therapy”) OR art therap* |
| 4. | (MH “Chiropractic”) OR chiropract* |
| 5. | (MH “Nutrition Services”) OR (dietician* OR dietitian* OR dietetic* OR nutritionist*) |
| 6. | physiotherap* |
| 7. | (MH “Physical Therapists”) OR physical therap* |
| 8. | (MH “Occupational Therapy”) OR occupational therap* |
| 9. | (MH “Social Work”) OR social work* |
| 10. | (MH “Music Therapy”) OR music therap* |
| 11. | (MH “Psychology”) OR psycholog* |
| 12. | (MH “Podiatry”) OR podiatr* |
| 13. | chiropod* |
| 14. | osteopath* |
| 15. | orthotist* |
| 16. | prosthetist* |
| 17. | speech therap* |
| 18. | speech patholog* |
| 19. | speech language therap* |
| 20. | oral health* |
| 21. | 1 or 2 or 3 or 4 or 5 or 6 or 7 or 8 or 9 or 10 or 11 or 12 or 13 or 14 or 15 or 16 or 17 or 18 or 19 or 20 |
| 22. | assistant* |
| 23. | support worker* |
| 24. | technician* |
| 25. | 22 or 23 or 24 |
| 26. | 21 and 25 |

**MH**: subject heading searched in EBSCO platform
