# Supplementary material for: The effect of delegation of therapy to allied health assistants on patient and organisational outcomes: a systematic review and meta-analysis
Source: BMC Health Serv Res. 2020 Jun 3;20:491. doi: 10.1186/s12913-020-05312-4 (PMC7268306; doi:10.1186/s12913-020-05312-4)
Supplement: Supplementary file 2 — Additional file 2. Minimum clinically important difference values. Minimum clinically important difference values used to determine clinical significance of findings [file 12913_2020_5312_MOESM2_ESM.docx]

Appendix 2. Minimum clinically important difference (MCID) values

| Outcome | MCID |
| --- | --- |
| Action Research Arm Test (units) | 12 [58] |
| Berg Balance Scale (units) | 7 [57] |
| Hospital Anxiety and Depression Scale: Depression Subscale (units) | 0.5 to 5.57 [54-56] |
| Pain – Visual Analogue Scale (cm) | 2.0 [53] |

Source provided in brackets.
