# Supplementary material for: The effect of delegation of therapy to allied health assistants on patient and organisational outcomes: a systematic review and meta-analysis
Source: BMC Health Serv Res. 2020 Jun 3;20:491. doi: 10.1186/s12913-020-05312-4 (PMC7268306; doi:10.1186/s12913-020-05312-4)
Supplement: Supplementary file 3 — Additional file 3. Study characteristics. Characteristics of the included studies [file 12913_2020_5312_MOESM3_ESM.docx]

Additional file 3. Study characteristics

| **Study**  **(design)** | **Therapy Delegated** | **Therapy Type** | **Participants**  **n** | **Study Setting**  **(country)** | **Outcomes** | |
| --- | --- | --- | --- | --- | --- | --- |
|  |  |  |  |  | **Patient** | **Organisation** |
| Boyle  2007 [46]  (RCT) | Group and individual language intervention | Substitution | Children aged 6-11 years with receptive or expressive language impairment  n=163 | Community  School-based  (Scotland) | Impairment | Cost effectiveness |
| Britton  2008 [27]  (RCT) | Sit-to-stand exercise | Additional | Diagnosis of stroke  n=18 | Sub-acute inpatient rehabilitation  (England) | Impairment  Activity limitation |  |
| Cannell  2018 [49]  (cohort) | Functional retraining exercise program | Substitution | Diagnosis of stroke  n= 73 | Sub-acute inpatient rehabilitation (AUS) | Activity limitation  Safety |  |
| Cox  2014 [50]  (cohort) | Group ADL retraining | Substitution | Aged care rehabilitation inpatients  n=70 | Inpatient Rehabilitation ward  (AUS) | Impairment  Activity limitation  Participation restriction  Other |  |
| Duncan  2006 [28]  (RCT) | Assist with feeding and provision of supplements | Additional | Woman aged ≥ 65 years with acute hip fracture  n=318 | Acute hospital trauma unit  (Wales) | Impairment  Safety  Other | Length of stay |
| **Study**  **(design)** | **Therapy Delegated** | **Therapy Type** | **Participants**  **n** | **Study Setting**  **(country)** | **Outcomes** | |
|  |  |  |  |  | **Patient** | **Organisation** |
| Hastings  2014 [29]  (cohort) | Walking exercise | Additional | Aged ≥ 65 years  n=127 | Acute hospital general medical unit  (USA) | Participation restriction  Safety | Length of stay  Hospital readmission |
| Howe  2005 [30]  (RCT) | Weight transference exercise | Additional | Diagnosis of stroke  n=35 | Acute hospital stroke unit  (England) | Activity limitation |  |
| Isbel  2014 [31]  (cohort) | Assist transition on discharge home | Additional | Aged ≥ 65 years  n=27 | Community  Home-based  (AUS) | Activity limitation |  |
| Jones  2006 [32]  (RCT) | Exercise program | Additional | Aged ≥ 65 years  n=160 | Acute hospital general medical unit  (AUS) | Activity limitation  Participation restriction  Safety | Length of stay |
| Lincoln  1999 [33] /Parry  1999 [34]  (RCT) | Upper limb exercise | Additional and substitution | Diagnosis of stroke (all)  n=188  mild impairment n=63 | Acute hospital OR sub-acute inpatient stroke unit  (England) | Impairment  Activity limitation |  |
| Lord  2008 [51]  (RCT) | Walking practice in community environment | Substitution | Diagnosis of stroke  n=36 | Community  Centre-based  (NZ) | Activity limitation  Participation restriction  Safety |  |
| **Study**  **(design)** | **Therapy Delegated** | **Therapy Type** | **Participants**  **n** | **Study Setting**  **(country)** | **Outcomes** | |
|  |  |  |  |  | **Patient** | **Organisation** |
| Niemela  2012 [35]  (cohort) | Exercise program | Additional | War veterans/ spouses  Female n=181  Male n=236 | Community  Home-based  (Finland) | Impairment  Activity limitation  Other |  |
| Nolan  2008 [36]  (cohort) | Exercise program | Additional | Aged ≥ 70 years  n=220 | Acute hospital general medical units  (AUS) | Activity limitation  Participation restriction | Length of stay  Hospital readmission |
| Parry  2016 [37]  (RCT) | Cognitive behavioural therapy | Additional | Aged ≥ 60 years with fear of falling  n=415 | Community  Home-based  (England) | Impairment  Activity limitation  Safety  Other | Cost effectiveness |
| Parsons  2018 [38]  (RCT) | Exercise program  ADL re-training | Additional | Aged ≥ 65 years discharged from an acute hospital ward  n=183 | Community  Home-based  (NZ) | Activity limitation | Length of stay  Hospital readmission |
| Pengas  2015 [39]  (cohort) | Exercise program | Additional | Post-elective arthroplasty  Hip n=470 knee  n=321 | Acute hospital orthopaedic unit  (Scotland) | Activity limitation | Length of stay |
| Salisbury  2010 [40]  (RCT) | Exercise program  Assist with feeding and provision of supplements | Additional | Discharged from an intensive care unit  n=16 | Acute hospital units  (Scotland) | Impairment  Activity limitation |  |
| **Study**  **(design)** | **Therapy Delegated** | **Therapy Type** | **Participants**  **n** | **Study Setting**  **(country)** | **Outcomes** | |
|  |  |  |  |  | **Patient** | **Organisation** |
| Shearer  2013 [41]  (cohort) | ADL retraining | Additional | Aged ≥ 65 years  n=48 | Acute hospital geriatric unit  (AUS) | Activity limitation  Participation restriction | Length of stay |
| Siebens  2000 [42]  (RCT) | Exercise program | Additional | Aged ≥ 70 years  n=300 | Acute hospital general medical and surgical units  (USA) | Activity limitation  Safety | Length of stay |
| Walsh  2015 [44]  (RCT) | Exercise program  Unspecified multi-disciplinary interventions | Additional | Discharged from an intensive care unit  n=240 | Acute hospital units  (Scotland) | Impairment  Activity limitation  Participation restriction  Safety  Other | Length of stay  Cost effectiveness |
| Weindling  2007 [43]  (RCT) | Exercise program | Additional | Children aged <4 years with cerebral palsy  n=57 | Community  Home-based  (England, Wales) | Impairment  Activity limitation | Cost effectiveness |
| Wenke  2014 [52]  (cohort) | Individual speech therapy | Substitution | Adults diagnosed with non-progressive neurological aphasia  n=17 | Inpatient rehabilitation unit  (AUS) | Activity limitation |  |

**ADL**: activities of daily living; **AUS**: Australia; **NZ:** New Zealand; **RCT**: randomised controlled trial; **USA**: United States of America. Note: Individual therapy (1:1) provided unless stated otherwise.
