# Supplementary material for: The effect of delegation of therapy to allied health assistants on patient and organisational outcomes: a systematic review and meta-analysis
Source: BMC Health Serv Res. 2020 Jun 3;20:491. doi: 10.1186/s12913-020-05312-4 (PMC7268306; doi:10.1186/s12913-020-05312-4)
Supplement: Supplementary file 4 — Additional file 4. Intervention characteristics: Additional AHA. Details of the additional AHA interventions for each study [file 12913_2020_5312_MOESM4_ESM.docx]

Additional file 4. Intervention characteristics: Additional AHA

| **Study** | **Setting** | **Profession Delegating** | **Intervention** | | **AHA Intervention** | | | | | | |
| --- | --- | --- | --- | --- | --- | --- | --- | --- | --- | --- | --- |
|  |  |  | **Usual Care** | **AHA** | **Program Duration** | **Session Frequency** | | **Session Duration** | | **Therapy Volume*** | |
| Britton  2008 [27] | Sub-acute inpatient rehabilitation | Physiotherapy | Routine physiotherapy and occ. therapy | Exercise program  (sit to stand mobility exercises) | 1 week | 5x/week | | 30 minutes | | 150 mins/week  Total: 150mins | |
| Duncan  2006 [28] | Acute hospital trauma unit | Dietetics | Routine provision of nutritional supplements | Provision of nutritional supplements and feeding aids, assistance with food choice, positioning and portion size, encouragement and assistance with feeding | Duration of hospital length of stay | n/s | | n/s | | n/s | |
| Hastings  2014 [29] | Acute hospital general medical unit | Physiotherapy | n/s | Exercise program (mobility exercises) | Duration of hospital length of stay | 5x/week | | 20 minutes | | 100 mins/week  Total: 500 mins | |
| Howe  2005 [30] | Acute hospital stroke unit | Physiotherapy | Routine physiotherapy | Exercise program (weight transference mobility, balance exercises) | 4 weeks | 3/week | | 30 minutes | | 90 mins/week  Total: 360 mins | |
| Isbel  2014 [31] | Community  Home-based | n/s | n/s | Assist transition home following hospital admission | n/s | n/s | | n/s | | n/s | |
| **Study** | **Setting** | **Profession Delegating** | **Intervention** | | **AHA Intervention** | | | | | | |
|  |  |  | **Usual Care** | **AHA** | **Program Duration** | | **Session Frequency** | | **Session Duration** | | **Therapy Volume*** |
| Jones  2006 [32] | Acute hospital general medical unit | Physiotherapy | Routine physiotherapy assessment and discharge planning | Exercise program (leg strength, abdominal strength, balance, mobility exercises) | Duration of hospital length of stay | | 10x/week | | 30 minutes | | 300 mins/week |
| Lincoln  1999 [33] /Parry [34] | Acute hospital OR sub-acute inpatient stroke unit | Physiotherapy | Routine physiotherapy | Upper limb exercise program (passive, assisted and active exercises; and functional exercises) | 5 weeks | | n/s | | n/s | | 120 mins/week  Total: 600 mins |
| Niemela  2012 [35] | Community  Home-based | Physiotherapy | Advice on healthy lifestyle and to continue usual daily activity | Exercise program (leg strength, balance, co-ordination exercises) | 10-14 months | | 1-2x/week | | 30 -60 minutes | | 30-60 mins/week |
| Nolan  2008 [36] | Acute hospital general medical OR aged care OR respiratory unit | Physiotherapy | Routine physiotherapy | Exercise program (leg strength, arm strength, balance, mobility exercises) | Duration of hospital length of stay | | 6x/week | | 30 minutes | | 180 mins/week |
| Parry  2016 [37] | Community  Home-based | Psychology | Falls prevention program consisting of: (1) Strength and balance exercise classes and (2) Home exercise program | Cognitive behavioural therapy for fear of falling | 8 weeks | | 1x/week | | 45 minutes | | 45 mins/week |
| **Study** | **Setting** | **Profession Delegating** | **Intervention** | | **AHA Intervention** | | | | | | |
|  |  |  | **Usual Care** | **AHA** | **Program Duration** | | **Session Frequency** | | **Session Duration** | | **Therapy Volume*** |
| Parsons  2018 [38] | Community  Home-based | Physiotherapy Occ. therapy | Discharge planning +/- community based services | Exercise program (mobility exercises) and ADL retraining | 6 weeks | | Up to 24x/week | | n/s | | n/s |
| Pengas  2015 [39] | Acute hospital orthopaedic unit | Physiotherapy | Routine orthopaedic physiotherapy | Exercise program  (exercises not specified) | Duration of hospital length of stay | | n/s | | n/s | | n/s |
| Salisbury  2010 [40] | Acute hospital units | Physiotherapy  Dietetics | Routine physiotherapy and dietetics | Exercise program (passive, active, strength exercises; balance, mobility exercises)  Provision of nutritional supplements and encouragement and assistance with feeding | Duration of hospital length of stay | | n/s | | n/s | | n/s |
| Shearer  2013 [41] | Acute hospital geriatric unit | Occ. therapy | Routine occ. therapy assessment and discharge planning | ADL re-training | Duration of hospital length of stay | | 3x/week | | 60 minutes | | 180 mins/week |
| Siebens  2000 [42] | Acute hospital general medical and surgical units | Physiotherapy | Routine hospital care | Exercise program (leg/arm flexibility, leg/arm strengthening, mobility exercises) | Duration of hospital length of stay | | 5x/week | | n/s | | n/s |
| **Study** | **Setting** | **Profession Delegating** | **Intervention** | | **AHA Intervention** | | | | | | |
|  |  |  | **Usual Care** | **AHA** | **Program Duration** | | **Session Frequency** | | **Session Duration** | | **Therapy Volume*** |
| Walsh  2015 [44] | Acute hospital units | Physiotherapy  Dietetics  Occ. therapy  Speech pathology | Routine physiotherapy, dietetics, occ. therapy and speech pathology | Exercise program (general exercise, balance, mobility exercises) and unspecified dietetic therapy, occ. therapy and speech therapy. | Duration of hospital length of stay | | n/s | | n/s | | n/s |
| Weindling  2007 [43] | Community  Home-based | Physiotherapy | Routine neuro-developmental physiotherapy | Exercise program (exercises not specified) | 6 months | | 1x/week | | 60 minutes | | 60 mins/week |

**AHA**: allied health assistant; **n/s**: not stated; **occ. therapy**: occupational therapy.

* Therapy volume refers to total volume each patient received.

Note: Individual therapy (1:1) provided unless stated otherwise.
