# Supplementary material for: The effect of delegation of therapy to allied health assistants on patient and organisational outcomes: a systematic review and meta-analysis
Source: BMC Health Serv Res. 2020 Jun 3;20:491. doi: 10.1186/s12913-020-05312-4 (PMC7268306; doi:10.1186/s12913-020-05312-4)
Supplement: Supplementary file 5 — Additional file 5. Intervention characteristics: Substitution AHA. Details of the substitution AHA interventions for each study [file 12913_2020_5312_MOESM5_ESM.docx]

Additional file 5. Intervention characteristics: Substitution AHA

| **Study** | **Setting** | **Program Duration** | **Intervention** | | | | |
| --- | --- | --- | --- | --- | --- | --- | --- |
|  |  |  | **AHA Description** | **AHP Description** | **Session Frequency** | **Session Duration** | **Therapy Volume*** |
| Boyle  2007 [46] | Community  school-based | 15 weeks | Group and individual speech therapy (monitoring, vocabulary, grammar and narrative) | **Speech Pathologist** Group and individual speech therapy (monitoring, vocabulary, grammar and narrative) | 3x/week | 30-40 minutes | 90-120 mins/week  Total: 1,350-1,800 mins |
| Cannell  2018 [49] | Sub-acute inpatient stroke unit | Duration of hospital length of stay | Individual functional retraining therapy including balance, upper/lower limb strength and endurance exercise | **Physiotherapist**  Group functional retraining therapy including balance, upper/lower limb strength and endurance exercise | 5x/week | 60 minutes | 300 mins/week |
| Cox  2014 [50] | Aged care inpatient rehabilitation unit | Duration of hospital length of stay | Group ADL retraining | **Occ. Therapist**  Group ADL retraining | Up to 6x/week | 60-120 minutes | n/s |
| Lincoln  1999 [33] /Parry [34]  1999 | Acute hospital OR sub-acute inpatient stroke unit | 5 weeks | Upper limb exercise program (passive, assisted and active exercises; and functional exercises) | **Physiotherapist**  Upper limb exercise program (facilitation, specific neuromuscular techniques and functional rehabilitation | n/s | n/s | 120 mins/week  Total: 600 mins |
| **Study** | **Setting** | **Program Duration** | **Intervention** | | | | |
|  |  |  | **AHA Description** | **AHP Description** | **Session Frequency** | **Session Duration** | **Therapy Volume*** |
| Lord  2008 [51] | Community  centre-based | 7 weeks | Exercise program (mobility practice in community environment) | **Physiotherapist** Exercise program (balance, motor relearning and mobility exercises) in health centre | 2x/week | n/s | n/s |
| Wenke 2014 [52] | Inpatient rehabilitation unit | 11 weeks | Individual speech therapy (auditory comprehension, reading comprehension, and written expression) | **Speech Pathologist** Group speech therapy (semantic-based, naming, verbal explanation and functional communication tasks) | 4-5x/week | 60-90 minutes | 240-450 mins/week  Total: 2,640-4,950 |

**AHA**: allied health assistant; **AHP**: allied health professional; **n/s**: not stated; **occ. therapist**: occupational therapist.

* Therapy volume refers to total volume each patient received.

Note: Individual therapy (1:1) provided unless stated otherwise.
