# Supplementary material for: The effect of delegation of therapy to allied health assistants on patient and organisational outcomes: a systematic review and meta-analysis
Source: BMC Health Serv Res. 2020 Jun 3;20:491. doi: 10.1186/s12913-020-05312-4 (PMC7268306; doi:10.1186/s12913-020-05312-4)
Supplement: Supplementary file 6 — Additional file 6. Downs and Black internal validity items. Study compliance with Downs and Black internal validity items [file 12913_2020_5312_MOESM6_ESM.docx]

Additional file 6. Downs and Black internal validity items

| **Study** | **Blinding** | | **Data** | **Analysis appropriate** | | **Intervention** | **Outcome** | **Group recruitment** | | **Group allocation** | | **Confounding factors taken into account** | |
| --- | --- | --- | --- | --- | --- | --- | --- | --- | --- | --- | --- | --- | --- |
|  | **Part** | **Assess** | **Data dredge clear** | **Adjust for length of follow up** | **Stat test selection** | **Reliable compliance** | **Reliable and valid** | **Same pop.** | **Same time** | **Random** | **Concealed** | **Confounding variables** | **Loss to follow up** |
| Boyle 2007 [46] | No | Yes | Yes | Yes | Yes | Yes | Yes | Yes | Yes | Yes | Yes | Yes | Yes |
| Britton 2008 [27] | No | No | Yes | Yes | Yes | Yes | Yes | Yes | Yes | Yes | Yes | No | No |
| Cannell  2018 [49] | No | Yes | Yes | Yes | Yes | Unable to determine | Yes | Yes | Yes | No | No | No | Yes |
| Cox  2014 [50] | No | No | No | No | Yes | Unable to determine | Yes | Yes | No | No | No | No | Yes |
| Duncan 2006 [28] | No | No | Yes | Yes | Yes | Unable to determine | Yes | Yes | Yes | Yes | Yes | Yes | Yes |
| Hastings 2014 [29] | No | No | Yes | No | Yes | No | Yes | Yes | Yes | No | No | No | Yes |
| Howe 2005 [30] | No | Yes | Yes | Yes | Yes | Yes | Yes | Yes | Yes | Yes | Unable to determine | Yes | No |
| Isbel 2014 [31] | No | No | Unable to deter-mine | Unable to deter-mine | Unable to determine | Unable to determine | Yes | Yes | No | No | No | Unable to determine | Unable to deter-mine |
| Jones 2006 [32] | No | Yes | Yes | No | Yes | Yes | Yes | Yes | Yes | Yes | No | Yes | Yes |
| **Study** | **Blinding** | | **Data** | **Analysis appropriate** | | **Intervention** | **Outcome** | **Group recruitment** | | **Group allocation** | | **Confounding factors taken into account** | |
|  | **Part** | **Assess** | **Data dredge clear** | **Adjust for length of follow up** | **Stat test selection** | **Reliable compliance** | **Reliable and valid** | **Same pop** | **Same time** | **Random** | **Concealed** | **Confounding variables** | **Loss to follow up** |
| Lincoln 1999 [33] / Parry 1999 [34] | No | Yes | Yes | Yes | Yes | Yes | Yes | Yes | Yes | Yes | Yes | Yes | Unable to deter-mine |
| Lord 2008 [51] | No | Yes | Yes | Yes | Yes | Yes | Yes | Yes | Yes | Yes | Yes | Yes | No |
| Niamela 2012 [35] | No | Yes | Yes | No | Yes | Unable to determine | Yes | No | Yes | No | No | No | Yes |
| Nolan 2008 [36] | No | No | Yes | No | No | Yes | Yes | Yes | Yes | No | No | Yes | No |
| Parry 2016 [37] | No | No | Yes | Yes | Yes | Yes | Yes | Yes | Yes | Yes | Yes | Yes | Yes |
| Parsons 2018 [38] | No | Yes | Yes | Yes | Yes | Unable to determine | Yes | Yes | Yes | Yes | Yes | Yes | Yes |
| Pengas 2015 [39] | No | No | Yes | No | Yes | Unable to determine | Unable to determine | Yes | Unable to deter-mine | No | No | Unable to determine | Unable to deter-mine |
| Salisbury 2010 [40] | No | Yes | Yes | Yes | Yes | Yes | Yes | Yes | Yes | Yes | No | No | No |
|  |  |  |  |  |  |  |  |  |  |  |  |  |  |
| **Study** | **Blinding** | | **Data** | **Analysis appropriate** | | **Intervention** | **Outcome** | **Group recruitment** | | **Group allocation** | | **Confounding factors taken into account** | |
|  | **Part** | **Assess** | **Data dredge clear** | **Adjust for length of follow up** | **Stat test selection** | **Reliable compliance** | **Reliable and valid** | **Same pop.** | **Same time** | **Random** | **Concealed** | **Confounding variables** | **Loss to follow up** |
| Shearer 2013 [41] | No | No | Yes | No | Yes | Unable to determine | Yes | Yes | No | No | No | Unable to determine | Unable to deter-mine |
| Siebens 2000 [42] | No | Yes | No | Yes | Yes | Yes | Yes | Yes | Yes | Yes | Yes | Yes | Yes |
| Walsh 2015 [44] | No | Yes | Yes | Yes | Yes | Yes | Yes | Yes | Yes | Yes | Yes | No | Yes |
| Weindling 2007  [43] | No | Yes | Yes | Yes | Yes | Yes | Yes | Yes | Yes | Yes | Yes | Yes | Yes |
| Wenke 2014 [52] | No | Unable to deter-mine | Yes | Yes | Yes | Yes | Yes | No | No | No | No | Unable to determine | No |

**Assess**: assessor; **Part**: participant; **Pop**: population; **Stat**: statistical.
